# Development and validation of the effective CNR analysis method for evaluating the contrast resolution of CT images

**DOI:** 10.1007/s13246-024-01400-5

**Published:** 2024-03-07

**Authors:** Kengo Igarashi, Kuniharu Imai, Shigeru Matsushima, Chiyo Yamauchi-Kawaura, Keisuke Fujii

**Affiliations:** Department of Integrated Health Sciences, Nagoya University Graduate School of Medicine, 1-1-20, Daiko-Minami, Higashi-ku, Nagoya, Aichi 461-8673 Japan

**Keywords:** Contrast resolution, Image noise, Contrast-to-noise ratio, Stochastic differential equation

## Abstract

Contrast resolution is an important index for evaluating the signal detectability of computed tomographic (CT) images. Recently, various noise reduction algorithms, such as iterative reconstruction (IR) and deep learning reconstruction (DLR), have been proposed to reduce the image noise in CT images. However, these algorithms cause changes in the image noise texture and blurred image signals in CT images. Furthermore, the contrast-to-noise ratio (CNR) cannot be accurately evaluated in CT images reconstructed using noise reduction methods. Therefore, in this study, we devised a new method, namely, “effective CNR analysis,” for evaluating the contrast resolution of CT images. We verified whether the proposed algorithm could evaluate the effective contrast resolution based on the signal detectability of CT images. The findings showed that the effective CNR values obtained using the proposed method correlated well with the subjective visual impressions of CT images. To investigate whether signal detectability was appropriately evaluated using effective CNR analysis, the conventional CNR analysis method was compared with the proposed method. The CNRs of the IR and DLR images calculated using conventional CNR analysis were 13.2 and 10.7, respectively. By contrast, those calculated using the effective CNR analysis were estimated to be 0.7 and 1.1, respectively. Considering that the signal visibility of DLR images was superior to that of IR images, our proposed effective CNR analysis was shown to be appropriate for evaluating the contrast resolution of CT images.

**Supplementary Information:**

The online version contains supplementary material available at 10.1007/s13246-024-01400-5.

## Introduction

X-ray computed tomography (CT) systems have progressed remarkably, and rapid data acquisition for obtaining high-resolution CT images is possible owing to the introduction of multiple-row detectors with thin element technology. As a result, CT examination offers numerous benefits in diagnosing several diseases and injuries, thus playing a significant role in diagnostic imaging. However, CT imaging is associated with higher radiation doses than conventional imaging modalities, raising concerns regarding the stochastic cancer risk associated with radiation exposure [1–3]. Because of the tradeoff between image quality and radiation dose, optimizing the radiation dose of radiographic imaging, including CT, is difficult. Therefore, obtaining CT images with low dose and high signal detectability is vital.

In general, three major factors play a significant role in the signal detectability of CT images: image noise, contrast, and sharpness. In particular, the detectability of low-contrast lesions, such as coronary plaques and hepatocellular carcinoma, is significantly affected by image noise. Thus, the development of noise reduction techniques is clinically useful. It is widely known that image noise can be statistically characterized by a Gaussian distribution [4, 5]. Several image noise reduction techniques have been devised based on this statistical characteristic, and one representative method is the iterative reconstruction (IR) algorithm. The IR algorithm has been implemented in most CT systems, making it possible to reduce image noise and improve signal detectability in CT images compared with the filtered back projection (FBP) algorithm [6]. However, some disadvantages of IR algorithms have been reported, such as strangeness owing to changes in the noise texture and blurring of lesion signals, which can lead to a misdiagnosis [7–11].

Recently, a new noise reduction algorithm using deep learning, called the deep learning reconstruction (DLR) algorithm, has been developed, and its performance has attracted considerable attention [12, 13]. DLR images have less image noise and demonstrate fewer changes in the noise texture than IR images [14]. However, no study has evaluated the signal detectability of DLR images, and the extent to which DLR algorithms improve the signal detectability of CT images remains debatable. Therefore, it is clinically important to investigate the signal detectability of DLR images.

The signal detectability of CT images is quantitatively evaluated using the contrast-to-noise ratio (CNR), which is the most popular evaluation index of contrast resolution. The CNR is defined as

1 $$CNR = \frac{{P_{signal} - P_{background} }}{{Noise~SD}},$$

where ${P}_{signal}$ is the average CT number of the signal region, ${P}_{background}$ is the average CT number of the background region around the signal, and $Noise SD$ is the standard deviation (SD) of the background region [5, 15]. This index is frequently used as a physical index for image quality control, performance comparison of modalities, optimization of scan and image processing conditions, and quantitative evaluation of lesion detectability because the image noise and contrast in Eq. (1) can be measured easily. However, IR and DLR algorithms are nonlinear processes that affect noise spatial correlations within an image and cause changes in noise texture and spatial resolution [16, 17]. Therefore, CNR evaluation is not consistent with visual assessment and is difficult to accurately perform. Thus, devising a new CNR analysis method that provides evaluation results similar to those of visual assessments is incredibly useful.

Therefore, in the present study, we proposed a new method for evaluating the contrast resolution of CT images, called the “effective CNR analysis method,” and verified its validity using CT images reconstructed using three noise reduction techniques.

## Methods

### CT image acquisition

**Fig. 1:**
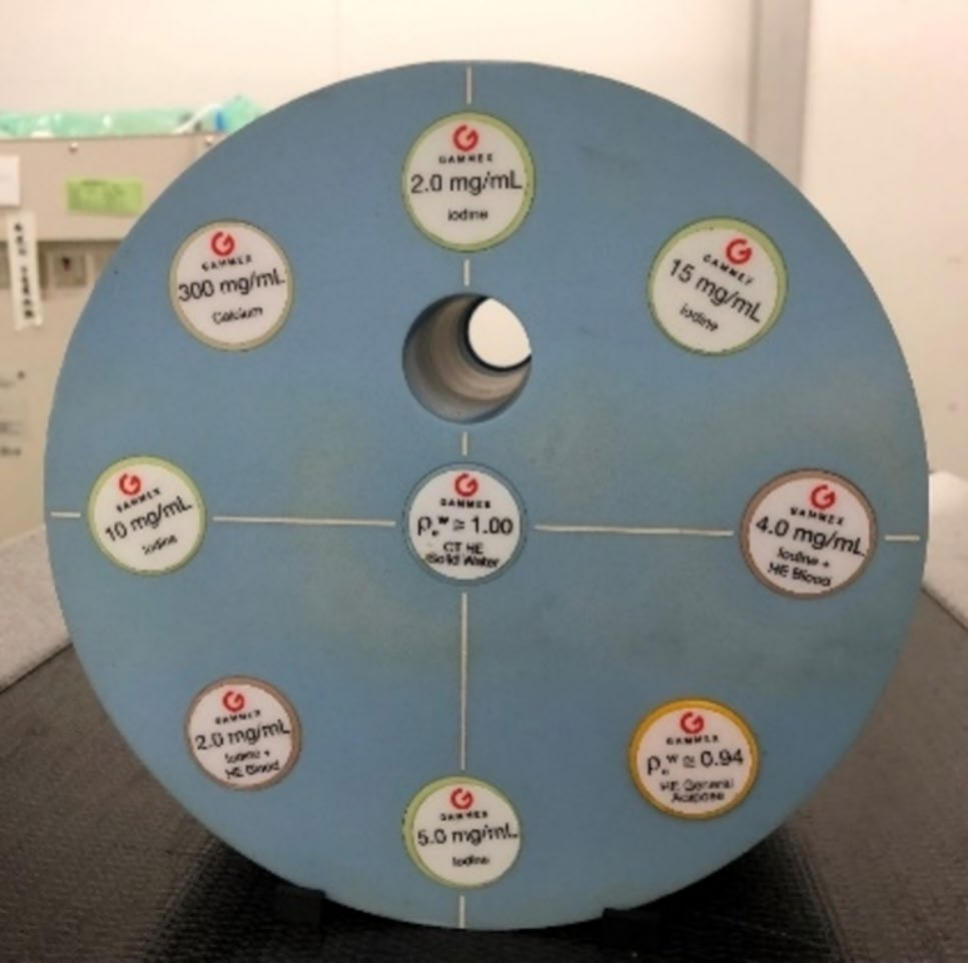
Multi-energy CT phantom

Figure 1 shows the target object, which is a commercially available phantom for quality control of CT images (Multi-energy CT phantom; Sun Nuclear Corporation, Florida). This phantom consisted of a cylinder made of a material equivalent to water and ten inserts made of different materials [18].

Currently, various algorithms for reconstructing CT images are installed in practical CT systems, enabling the detection of subtle (low-contrast) lesions. However, detectability is considered to differ depending on the reconstruction algorithm. Thus, it is useful to investigate the effects of reconstruction algorithms on signal detectability. Furthermore, when a subtle lesion cannot be detected, contrast-enhanced CT examinations are performed, which play a significant role in the diagnostic imaging of the lesions. In this study, iodine- and blood-equivalent modules in the phantom were used as high- and low-contrast target objects, respectively, to evaluate signal detectability.

The phantom was scanned using a 320-row area detector CT (Aquilion ONE/PRISM Edition; Canon Medical Systems Corporation, Japan), and the scan parameters are listed in Table 1. CT images were reconstructed with a slice thickness of 5 mm using three image reconstruction algorithms: FBP, model-based IR (Forward projected model-based Iterative Reconstruction SoluTion [FIRST] [19]), and DLR (Precise IQ Engine [PIQE]). PIQE, released in Canon Medical’s CT system, is a new image reconstruction algorithm developed for cardiac CT images [13]. Therefore, a cardiac kernel was used for FIRST and PIQE in this study.

**Table 1:** CT scan and image reconstruction parameters

Scan parameter	Conditions
Tube Voltage (kVp)	80
Tube Current (mA)	100
Exposure time (sec / rotation)	0.5
Beam collimation (mm)	40
Scan mode	Non-Helical
Reconstruction algorithm (Kernel, Strength)	FBP (FC13, None) FIRST (Cardiac, Standard) PIQE (Cardiac, Standard)
Slice thickness (mm)	5
Display field-of-view (mm)	320 (Original field-of-view) 100 (Extended field-of-view)

**Fig. 2:**
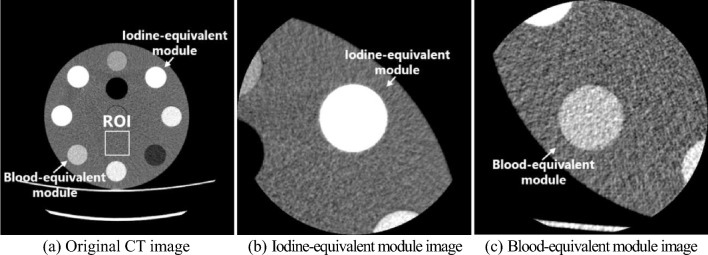
Original CT image and CT images using extended field-of-view reconstruction

 Figure 2 shows a phantom CT image for CNR analysis. As shown in Fig. 2a, to evaluate image noise, a square region of interest (ROI) of 40 × 40 pixels was placed at the center of the phantom CT images. Furthermore, CT images of iodine- and blood-equivalent modules shown in Fig. 2b and c were employed as target images for evaluating image contrast and were acquired using extended field-of-view reconstruction to accurately measure edge profiles. In the present study, the image noise and edge profile were evaluated using the analysis method, which is explained in the following sections based on these figures. Moreover, a quantitative evaluation of contrast resolution using the proposed method was performed for CT images reconstructed using three types of reconstruction algorithms. For comparison, contrast resolution was also evaluated using the conventional method.

### Effective CNR analysis

In the “effective CNR analysis” proposed in this study, an evaluation method for image noise by considering the noise texture and that for contrast by considering the sharpness in CT images are necessary. The evaluation methods are described as follows:

### Evaluation of apparent noise on CT images

#### Principle

Image noise on CT images can be characterized by its magnitude and texture [20]. Noise magnitude refers to random fluctuations of CT numbers within a homogenous ROI and is expressed as the SD of CT numbers within the ROI (noise SD). Meanwhile, noise texture refers to correlations between adjacent CT numbers that are manifested by the grainy appearance of CT images. CT images with equal noise magnitudes but different noise textures may not have the same image quality [21]. IR and DLR algorithms are nonlinear processes that affect noise spatial correlations within an image and, thus, cause changes to the noise texture and spatial resolution [16, 17]. In particular, the appearance of noise texture, such as blocky noise, is recognized on CT images scanned at low radiation doses and reconstructed using IR algorithms; hence, the noise texture affects the detection of low-contrast lesions [22–24]. Therefore, the noise evaluation of CT images requires the analysis of both the noise and texture. Fujii et al. devised a simple method for evaluating noise texture (apparent noise), such as the blocky noise observed in low-dose CT images reconstructed using the nonlinear IR and DLR algorithms, and concluded that the apparent noise index measured using this method is valid and useful as an indicator to quantify and compare the noise texture of the CT images obtained with different scan parameters and reconstruction algorithms [25]. In the present study, the apparent noise index was employed as a physical index for evaluating image noise in CT images.

#### Measurement

**Fig. 3:**
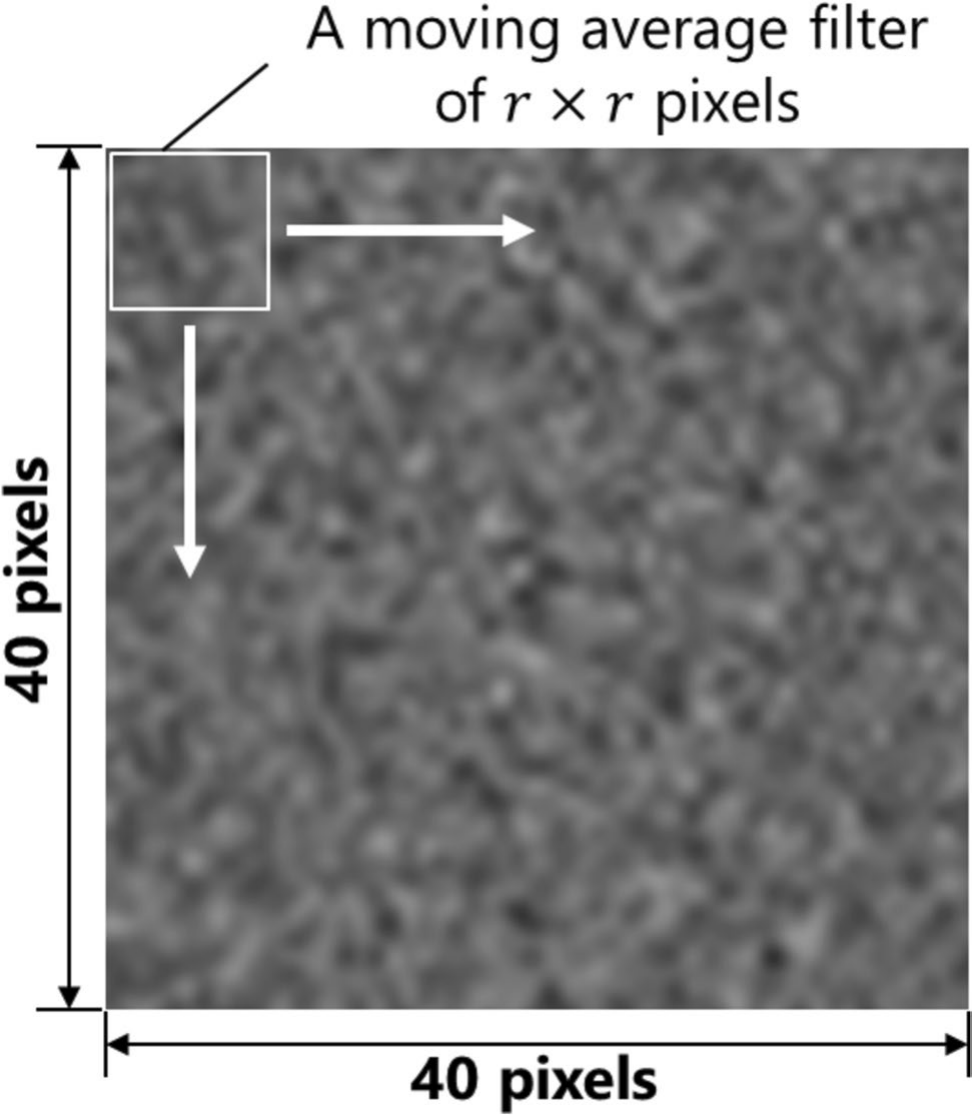
Moving average filter in ROI

Figure 3 shows an enlarged image of the ROI in Fig. 2a. To evaluate the image noise, we applied a moving average filter with a pixel size of $r\times r$ (*r* = 1, 2, …, 20) to the ROI, sliding the filter vertically and horizontally in increments of 1 pixel, as shown in Fig. 3, and the SD of the mean CT numbers for each filter size was calculated. In the report by Fujii et al. [25], using a log–log scale, the SD of the mean CT numbers exhibited a strong linear relationship with filter sizes ranging from $5\le r\le 10$ pixels and could be fitted with the curve shown in Eq. (2) because of the central limit theorem.

2 $$\sigma _{{\text{SD}}} = \frac{{\sigma _{{\text{Apparent}}} }}{r},$$

where ${\sigma }_{SD}$, ${\sigma }_{Apparent}$, and $r$ represent the SD of the mean CT numbers in each moving average filter, apparent noise index, and length of one side of the filter, respectively.

### Evaluation of contrast by considering the sharpness on CT images

#### Principle

Currently, contrast in CT images is defined only as the difference between the average CT numbers of the signal and the adjacent background regions (hereafter referred to as “contrast”). However, when diagnostic imaging is performed using CT, identifying the location, size, and shape of the lesions requires high accuracy. Therefore, it is important to depict signal contours clearly. CT images reconstructed using noise reduction algorithms have reduced sharpness, and their visual impressions may differ even if the contrast is the same [10]. Therefore, devising a method for evaluating the contrast that considers sharpness on CT images is clinically important.

**Fig. 4:**
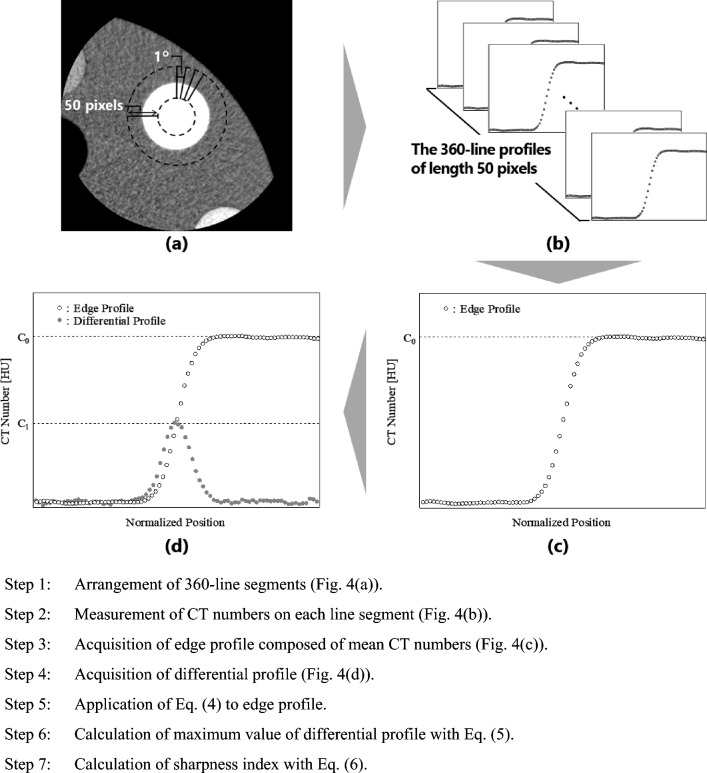
Derivation process of sharpness index

Rüdinger and Spiegler devised a sharpness evaluation method for diagnostic X-ray images based on the shape of the signal profile [26]. The sharpness index is given as the ratio between the signal intensity of the edge profile and that of the slit profile and is defined as

3 $$Sharpness~Index = \frac{{C_1 }}{{C_0 }},$$

where ${C}_{0}$ and ${C}_{1}$ represent the signal intensities of the edge and slit profiles, respectively. As shown in Fig. 4, the contrast and maximum values of the differential profile obtained from the edge profile can be regarded as the intensities of the edge and slit profiles, respectively. Therefore, the sharpness index can be applied as a physical indicator to evaluate the CT image quality. As shown in Eq. (3), if the signal of CT images is not blurred, the intensities ${C}_{0}$ and ${C}_{1}$ remain the same; however, if the signal is blurred, ${C}_{1}$ decreases. In other words, the sharpness index decreases as the blurring of the CT image increases. In this study, we proposed a contrast evaluation method that considers the sharpness of CT images by defining the effective contrast as the image contrast multiplied by the sharpness index.

#### Measurement

Figure 4 shows the derivation of the sharpness index. The 360-line segments, each of 50-pixel length, were placed in the radial direction at a central angle interval of 1° (Fig. 4a), and the CT numbers on each line segment were measured (Fig. 4b). Subsequently, the mean CT numbers were calculated from the CT numbers at the pixels located at the same distance from the image signal center (Fig. 4c). Thus, the edge profile used in this analysis was composed of 50 mean CT numbers. Furthermore, to calculate the sharpness index using Eq. (3), the slit profile should be deduced. Therefore, this profile, termed the “differential profile,” was obtained by subtracting two adjacent mean CT numbers in the edge profile (Fig. 4d). However, it is difficult to measure the edge profile accurately because it contains image noise. Previously, Imai et al. reported that by modeling the edge profiles of CT images using stochastic differential equations, a formula for the estimated edge profile as a solution to the equation was derived and is expressed as follows [27]:

4 $$\begin{aligned} f\left( {X_{\left( t \right)} ,~t} \right) = & C_0 \mathop \smallint \limits_{ - \infty }^{g\left( t \right)} \frac{1}{{\sqrt {{2\pi }} }}e^{\frac{{\varsigma ^2 }}{2}} d\varsigma = C_0 \phi \left( {g\left( t \right)} \right) \\ g\left( t \right) = & \frac{1}{{\sigma \sqrt {{T - t}} }}\left[ {\lambda + \left( {\gamma + \frac{{\sigma ^2 }}{2}} \right)\left( {T - t} \right)} \right], \\ \end{aligned}$$

where ${X}_{\left(t\right)}$ is a CT number at any position in the CT image, ${C}_{0}$ is the contrast, $\phi \left(g\right({X}_{\left(t\right)},t\left)\right)$ is the blur function, $\sigma$ is the diffusion coefficient, $T$ is the profile length, $\lambda$ is $\text{ln}{10}^{-13}$, and $\gamma$ is the variable associated with the shape of the edge profile (details are explained in Supplementary Information) [27]. In this study, using Eq. (4), we obtained the estimated edge profile of the signal in CT images based on the measured edge profile under the following conditions: length of the edge profile $T=50$ pixels and sampling interval $\varDelta t=0.10$. Thus, we obtained the contrast ${C}_{0}$ from the formula of the estimated edge profile and determined the differential profile as follows:

5 $$\frac{{df\left( {X_{\left( t \right)} ,~t} \right)}}{{dt}} = C_0 \frac{{d\phi \left( {g\left( t \right)} \right)}}{{dt}}.$$

Given that ${C}_{1}$ in Eq. (3) is the maximum value of Eq. (5), the sharpness index can be expressed as

6 $$Sharpness~Index = \frac{{\max \left( {\frac{{df\left( {X_{\left( t \right)} ,t} \right)}}{{dt}}} \right)}}{{C_0 }} = \max \left( {\frac{{d\phi \left( {g\left( t \right)} \right)}}{{dt}}} \right).$$

Therefore, the effective contrast of the CT image to be analyzed was obtained by multiplying the sharpness index with the image contrast. This is expressed in Eq. (7) as follows:

7 $$\begin{aligned} Contrast_{blur} = & Contrast \times Sharpness~Index \\ = & C_0 \times \max \left( {\frac{{d\phi \left( {g\left( t \right)} \right)}}{{dt}}} \right). \\ \end{aligned}$$

### Effective CNR

We defined and explained the evaluation methods of “apparent noise” and “contrast by considering the sharpness” in the preceding sections. Herein, using these physical indices, we defined the “effective CNR” as follows:

8 $$Effective~CNR = \frac{{C_0 \times \max \left( {\frac{{d\phi \left( {g\left( t \right)} \right)}}{{dt}}} \right)}}{{\sigma _{Apparent} }}.$$

Using this formula, we evaluated the effective CNR of analytical CT images.

## Results

**Fig. 5:**
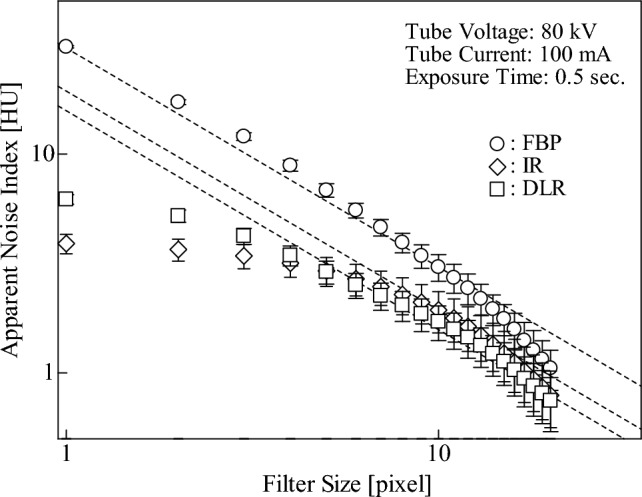
${\sigma }_{SD}$ of the FBP, IR, and DLR images as a function of the filter size

Figure 5 shows the ${\sigma }_{SD}$ values of each moving average filter on FBP, IR, and DLR images as a function of the filter size (one side length). Regardless of the image type, ${\sigma }_{SD}$ decreased for a filter size of ≥ 5 pixels. In particular, we confirmed that ${\sigma }_{SD}$ for filter sizes ranging from $5\le r\le 10$ pixels can be fitted with the curve (dashed line in the figure) shown in Eq. (2), as reported by Fujii et al. [25]. Thus, the apparent noise evaluation method can be applied to the images analyzed in this study. The ${\sigma }_{Apparent}$ values of FBP, IR, and DLR images were 30.45 HU, 17.82 HU, and 15.76 HU, respectively.

**Fig. 6:**
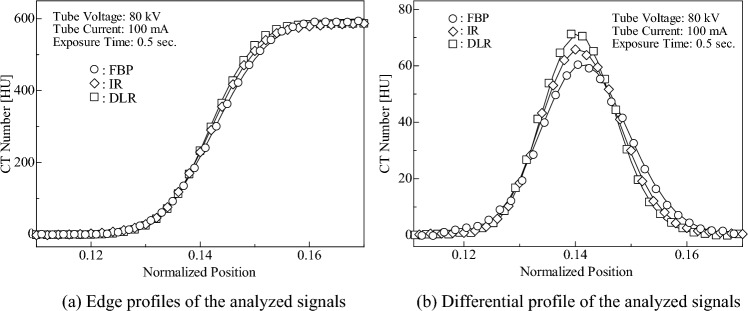
Profiles of the iodine-equivalent module

Figure 6 shows the edge and differential profiles of the iodine signal image reconstructed using the three reconstruction algorithms. The solid lines in the figures represent the curves estimated using Eq. (4), where each plot represents the CT number of the mean CT number profile at the position normalized by the profile length. The mean CT numbers lie on the curve generated using Eq. (4), regardless of the reconstruction algorithm used. Therefore, the edge profile was governed by Eq. (4). Furthermore, as illustrated in Fig. 6a, the image contrasts showed little difference among the CT images reconstructed using three kinds of reconstruction algorithms, whereas in FBP and IR images, the slopes of the edge area were slightly more gradual. In line with this variation, their differential profiles showed a wider tail and a lower maximum value than those of the DLR image, as shown in Fig. 6b. Given these results, the image contrast of the CT image for each reconstruction algorithm was evaluated using Eq. (7). Consequently, the image contrasts for FBP, IR, and DLR were estimated to be 60.31 HU, 65.79 HU, and 75.16 HU, respectively. These results indicate that for the iodine signal, the DLR images had high image contrast when considering sharpness compared with the FBP and IR images.

**Fig. 7:**
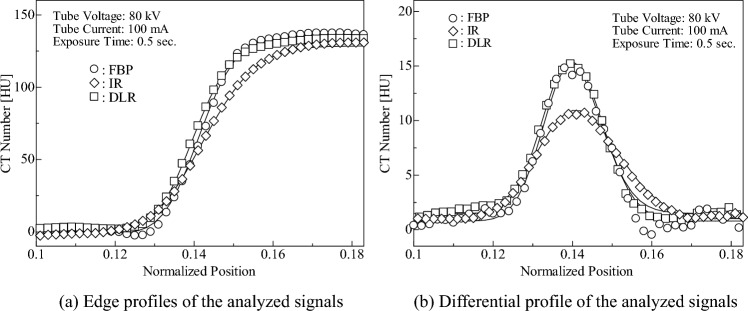
Profiles of the blood-equivalent module

The edge and differential profiles of the blood-signal image are shown in Fig. 7. As illustrated in Fig. 7a, although the image contrast is low, the edge profile can be estimated using Eq. (4). Therefore, the edge profile was shown to be governed by the theoretical formula derived from the stochastic differential equation for CT numbers, regardless of the reconstruction algorithms and image contrasts. Furthermore, in the evaluation of the image contrast based on Eq. (7), DLR yielded the highest image contrast among the three kinds of reconstruction algorithms, as with iodine image signals (15.01 HU for FBP, 12.02 HU for IR, and 16.64 HU for DLR).

**Fig. 8:**
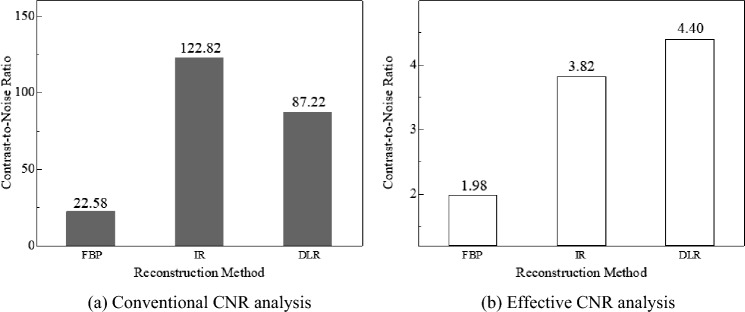
Results of the CNR analyses of iodine-equivalent module

**Fig. 9:**
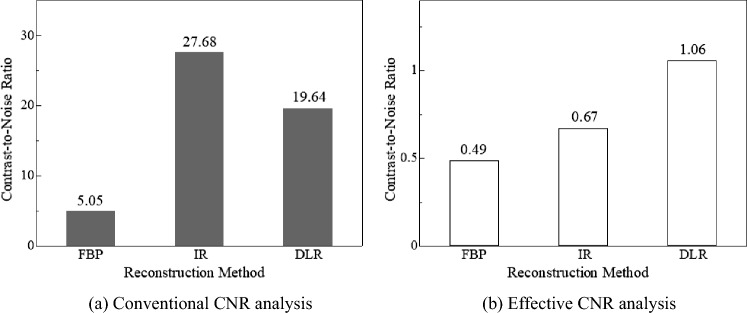
Results of the CNR analyses of blood-equivalent module.

**Fig. 10:**
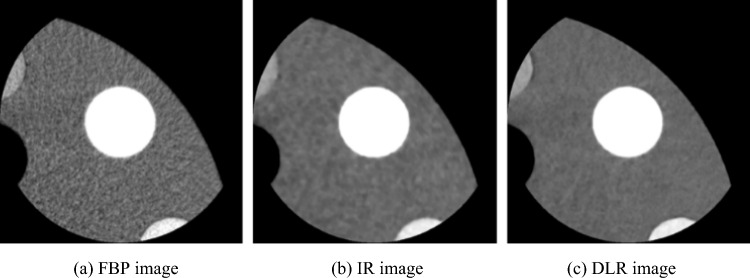
CT images of iodine-equivalent module reconstructed using three noise reduction algorithms (Window level: 40 HU, Window width: 400 HU)

**Fig. 11:**
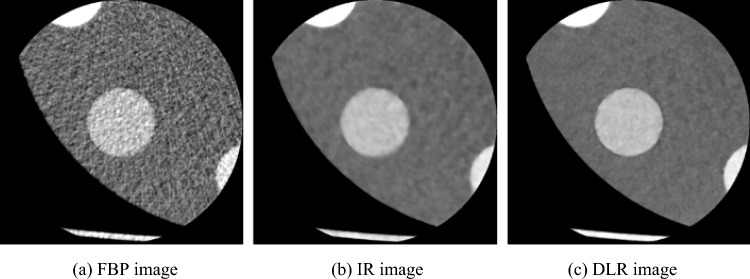
CT images of blood-equivalent module reconstructed using three noise reduction algorithms (Window level: 40 HU, Window width: 400 HU)

 Figures 8 and 9 show the CNR evaluation of the signal images reconstructed using the three reconstruction algorithms. As shown in Fig. 8a, in the CNR evaluation of iodine signal images with the conventional CNR, the IR images had a high CNR compared to the other two CT images. In contrast, as shown in Fig. 8b, in CNR evaluation using effective CNR, the DLR images showed slightly higher values than IR images, and FBP images showed the lowest CNR in both CNR evaluations. When subjectively comparing the FBP, IR, and DIR images, the sharpness of the signal in the IR image was reduced, and the DLR images showed higher signal visibility, as illustrated in Fig. 10. This finding was obtained for both iodine- and blood-signal images. In other words, effective CNR yielded different results from those of conventional CNR, as shown in Fig. 9. Furthermore, a comparison of Figs. 9 and 11 indicates that CNR evaluation using effective CNR is consistent with the subjective assessment. These results suggest that the proposed effective CNR analysis is appropriate for evaluating the contrast resolution of CT images.

## Discussion

In this study, we adopted apparent noise as the image noise index and contrast by considering sharpness as the contrast index to develop an effective CNR analysis method for evaluating the contrast resolution of CT images.

Similar to that reported by Fujii et al. [25], in the present study, we showed that the ${\sigma }_{SD}$ of FBP images decreased with filter size; the ${\sigma }_{SD}$ of IR and DLR images decreased for filter sizes with ≥ 5 pixels; and the ${\sigma }_{SD}$ variation with filter sizes with $5\le r\le 10$ pixels can be fitted with the curve shown in Eq. (2). This means that ${\sigma }_{Apparent}$ can be easily calculated by incorporating a filter size of $5\le r\le 10$ pixels (e.g., *r* = 7) and its ${\sigma }_{SD}$ into Eq. (2) without drawing a graph, as shown in Fig. 5. The ${\sigma }_{Apparent}$ values of FBP, IR, and DLR images were 30.45 HU, 17.82 HU, and 15.76 HU, respectively, which were higher than conventional noise SDs (FBP: 30.28 HU, IR: 4.81 HU, and DLR: 6.79 HU). However, ${\sigma }_{Apparent}$ of FBP images was similar to that of noise SD, corresponding to the ${\sigma }_{SD}$ when $r=1$ in Eq. (2). By contrast, the ${\sigma }_{Apparent}$ values of IR and DLR images were significantly larger than their noise SDs. These results indicate that the noise SD cannot be used to evaluate the image noise, including noise texture, leading to an underestimation. As shown in the CT images in Figs. 10 and 11, the DLR image had a more uniform background area without any change in noise texture compared to the IR image. Therefore, the evaluation method for apparent noise in CT images can be used to determine the noise texture and statistical noise, such as quantum or electrical noise, and yield results similar to those of subjective recognition.

Furthermore, we showed that DLR images had the highest contrast, considering the sharpness of the analyzed CT images. In this study, PIQE was used as the DLR algorithm. PIQE uses a deep learning neural network and is trained with ultra-high resolution CT data, which have a matrix size of 0.25 mm, to maximize the inherent spatial resolution of a conventional CT image, which has a matrix size of 0.5 mm, and then enhance it. This is referred to as the super-resolution algorithm [13]. In other words, PIQE images may be less affected by partial volume effects than CT images reconstructed using the conventional FBP or IR methods. To verify the validity of the contrast evaluation by considering sharpness, a subjective evaluation was conducted, as shown in Figs. 10 and 11, similar to the case of apparent noise evaluation. DLR images had a more obvious signal contour than FBP and IR images. This result is consistent with that reported in a previous study, where PIQE images exhibited improved signal visibility [28]. In view of these results, the contrast evaluation method that considers sharpness in CT images is valid. Thus, this analysis method is potentially superior to the traditional quantitative technique.

**Fig. 12:**
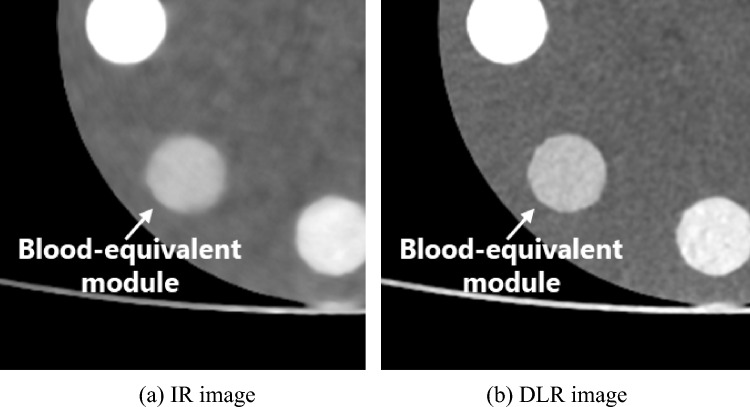
Difference in subjective assessment of signals between IR and DLR images (Window level: 40 HU, Window width: 400 HU)

 The CNRs of the DLR images were higher than those of the IR images in the effective CNR analysis. However, the CNRs of the IR images were higher in the conventional CNR analysis. Two types of CT images were prepared to verify the validity of the evaluation (Fig. 12). Figure 12a, b show the IR and DLR images, respectively. This figure clearly shows that the signal visibility of the DLR images is superior to that of the IR images. CNR analysis was performed on these figures using both the proposed and conventional CNR analysis methods. The CNRs of the IR and DLR images calculated using the conventional CNR were 13.2 and 10.7, respectively, whereas those calculated using the effective CNR were 0.7 and 1.1, respectively. Based on these CNR evaluations, the DLR images were shown to have a higher contrast resolution than the IR images. Additionally, for subjective recognition, which was also performed for Figs. 10 and 11, the signal detectability of the DLR image was shown to be higher than that of the IR image. The reason for obtaining these evaluations may be attributed to the results of the noise and contrast evaluations, which were similar to the results of subjective recognition. Therefore, our proposed effective CNR is a useful physical index for evaluating contrast resolution, and the signal detectability of CT images cannot be properly evaluated without considering the noise SD, contrast, noise texture, and sharpness.

This study had some limitations. We did not compare the evaluation of signal detectability using the proposed analysis method with the subjective evaluation of image quality—such as the evaluation of noise, sharpness, and contrast—with the Likert scale. These visual evaluations are important for validating the reliability of the proposed analysis method. In addition, the target images used in this study for analyzing signal detectability were phantoms and not clinical images. To further validate the usefulness of the proposed analysis method, clinical images must be used as target images. In practice, it is important to evaluate the detectability of lesions, such as small coronary plaques and hepatocellular carcinoma. Therefore, our proposed effective CNR analysis method must be applied to the quantitative assessment of image quality in clinical CT images and compared with lesion detection by radiologists. These challenges will be addressed in future research.

## Conclusion

In this study, we devised a new quantitative evaluation method for contrast resolution, termed the “effective CNR analysis method,” with CT images reconstructed using the FBP, IR, and DLR methods. The results are summarized as follows:

1. Using the apparent noise index, we evaluated image noise based on noise intensity and texture.
2. Depending on the type of image reconstruction method, even if the signal area to be analyzed is the same, its edges are blurred, and the contrast value when considering sharpness is reduced.
3. The effective CNR analysis method could be applied to CT images as potentially superior to the traditional quantitative technique.

These results indicated that the effective CNR analysis method was valid. Furthermore, this analysis quantitatively demonstrated that DLR images have a better contrast resolution than IR images. The proposed method comprehensively evaluates image noise, contrast, and sharpness, which are largely related to signal detectability. We believe that this is a useful quantitative evaluation index for examining the signal detectability of CT images.

### Supplementary Information

Below is the link to the electronic supplementary material.

Supplementary material 1 (DOCX 23 kb)
