# Supplementary material for: Development and validation of the effective CNR analysis method for evaluating the contrast resolution of CT images
Source: Phys Eng Sci Med. 2024 Mar 7;47(2):717–27. doi: 10.1007/s13246-024-01400-5 (PMC11166862; doi:10.1007/s13246-024-01400-5)
Supplement: Supplementary file 1 — Supplementary material 1 (DOCX 23 kb) [file 13246_2024_1400_MOESM1_ESM.docx]

**Development and validation of the effective CNR analysis method for evaluating the contrast resolution of CT images**

Kengo Igarashi^1^, Kuniharu Imai^1^, Shigeru Matsushima^1^, Chiyo Yamauchi-Kawaura^1^, Keisuke Fujii^1^

^1^Department of Integrated Health Sciences, Nagoya University Graduate School of Medicine, 1-1-20, Daiko-Minami, Higashi-ku, Nagoya, Aichi 461-8673, Japan

***Corresponding author: Kengo Igarashi. Email: igarashi.kengo.a5@s.mail.nagoya-u.ac.jp**

**Appendix**

**Estimated equation for edge profiles on CT images based on the stochastic differential equation**

Deriving the estimated equation for edge profiles on CT images based on the stochastic differential equation has been reported only in Japanese [27]; we will briefly explain its derivation here.

Medical images such as CT images can be modeled as the sum of deterministic and stochastic factors. CT images have stochastic factors that depend on the CT numbers, such as blur caused by scattered X-rays and image noise caused by variations in X-ray photons. Given these considerations, the process of obtaining CT images can be considered as a Geometric Brownian motion or the Wiener process and can be formulated by the stochastic differential equation as,

$$dX_{(t)}=\alpha X_{(t)}dt+\sigma X_{\left( t \right)}dw ・・・(1)$$

where $X_{(t)}$ is the CT number at any position in the CT image, $\alpha$ is the drift coefficient, $\sigma$ is the diffusion coefficient, and *w* is the Wiener process. On the right side of Eq. (1), the first and second terms represent the deterministic and stochastic factors, respectively.

In general, the sharpness of X-ray images is evaluated using edge profiles or slit profiles approximated by the exponent and Gaussian functions to eliminate image noise and sampling error. Therefore, the CT numbers in the edge profile are considered to change exponentially. Given that the edge profile $f\left( X, t \right)$ follows the Winner process, using Itô’s lemma, Eq. (1) can be modified as follows:

$$\frac{\partial f\left( X_{(t)}, t \right)}{\partial t}+\frac{1}{2}\sigma^{2}X_{(t)}^{2}\frac{\partial^{2}f(X_{(t)}, t)}{\partial X_{(t)}^{2}}+rX_{(t)}^{2}\frac{\partial f(X_{(t)}, t)}{\partial X_{(t)}}-rf\left( X_{\left( t \right)}, t \right)=0 ・・・\left（ 2 \right）$$

where **r** is the variable associated with the shape of the edge profile. As shown, by applying Ito’s lemma, the second term, which is a stochastic factor in Eq. (1), is eliminated, resulting in the following second-order partial differential equation. Let $T$ be the length of the edge profile and, under the condition that the CT number at $t=T$ is $X_{(t)}=X_{0}$, $f\left( X_{(t)}, t \right)$ is given as follows:

$$f\left( X_{(t)}, t \right)=e^{r\left( t-T \right)}u\left( X_{\left( t \right)}, t \right). ・・・(3)$$

Considering Eq. (3), Eq. (2) can be transformed as,

$$\frac{\partial u\left( X_{(t)}, t \right)}{\partial t}+rX_{(t)}\frac{\partial u(X_{(t)}, t)}{\partial X_{(t)}}+\frac{1}{2}\sigma^{2}X_{(t)}^{2}\frac{\partial^{2}u(X_{(t)}, t)}{\partial X_{(t)}^{2}}=0. ・・・(4)$$

Additionally, variable transformation was performed as follows:

$$y=\ln X_{0}+\left( r-\frac{1}{2}\sigma^{2} \right)\left( T-t \right)$$

$$s=T-t$$

such that $u\left( X_{(t)}, t \right)$ becomes v$\left( y, s \right)$, resulting in following equation:

$$-\frac{\partial v\left( y, s \right)}{\partial s}+\frac{1}{2}\sigma^{2}\frac{\partial^{2}v\left( y, t \right)}{\partial y^{2}}=0. ・・・(5)$$

To simplify this equation, let us consider the variable $y$ as $z$, where *z* is expressed as,

$$z=\frac{y}{\sigma} ,$$

we obtained the transformed equation as follows:

$$\frac{\partial w(z, s)}{\partial s}=\frac{1}{2}\frac{\partial^{2}w(z, s)}{\partial z^{2}}. ・・・\left（ 6 \right）$$

This equation is known as the diffusion equation. From this equation, we found that the edge profiles on the CT images are governed by a diffusion equation. This finding indicates that the CT images are blurred by the Compton effect. Therefore, Eq. (6) is a reasonable equation for evaluating the sharpness of CT images, and its solution reflects the sharpness of the CT images. Generally, the fundamental solution to the diffusion equation is a Gaussian distribution. Let $w\left( z, 0 \right)$ be explained by the following equation as the initial condition of Eq. (6), under the condition that the CT number at $t=T$ is $X_{(t)}=X_{0}$:

$$w\left( z, 0 \right)=e^{\sigma z} ,$$

a solution of Eq. (6) can be represented as,

$$w\left( z, s \right)=e^{\sigma z}\int_{-\infty}^{\infty} \frac{1}{\sqrt{2\pi s}}e^{\frac{-{(z-\xi)}^{2}}{2s}}d\xi・・・(7)$$

where $\xi$ is the variable. This indicates that $u\left( X_{(t)}, t \right)$ in Eq. (3) is a Gaussian function, and the edge profile $f\left( X_{(t)}, t \right)$ consists of an exponential function and a Gaussian function. To determine the specific edge profile $f\left( X_{(t)}, t \right)$, we performed a reverse manipulation of the manipulations we previously performed. Using the cumulative distribution of the standard normal distribution, $f\left( X_{(t)}, t \right)$ is represented as,

$$f\left( X_{(t)}, t \right)=C_{0}\int_{-\infty}^{g(X_{(t)}, t)} \frac{1}{\sqrt{2\pi}}e^{\frac{\varsigma^{2}}{2}}d\varsigma=X_{0}\phi\left( g(X_{(t)}, t) \right)$$

$$g\left( X_{(t)}, t \right)=\frac{1}{\sigma\sqrt{T-t}}\left[ \ln X_{0}+\left( r+\frac{\sigma^{2}}{2} \right)\left( T-t \right) \right].・・・(8)$$

Although it is known that the sharpness of X-ray images depends on the object contrast but not on the image contrast (as shown in Eq. (8)), $g\left( X_{(t)}, t \right)$, which is associated with the shapes of edge profiles, depends on $X_{0}$. As the fact that $X_{0}$ for $X_{0}<{10}^{-9}$ is independent of $\phi\left( g(X_{(t)}, t) \right)$ has been experimentally demonstrated, the edge profiles were deduced under $X_{0}={10}^{-13}$ according to reference 27.
